# The risk factors for *Group B Streptococcus* colonization during pregnancy and influences of intrapartum antibiotic prophylaxis on maternal and neonatal outcomes

**DOI:** 10.1186/s12884-023-05478-9

**Published:** 2023-03-27

**Authors:** Xiaoli Chen, Sijia Cao, Xiaochun Fu, Yan Ni, Bixuan Huang, Jiayin Wu, Ling Chen, Shuying Huang, Jiali Cao, Weiwei Yu, Huiming Ye

**Affiliations:** 1 Department of Clinical Laboratory, Women and Children’s Hospital, School of Medicine, Xiamen University, Xiamen, China; 2 School of Basic Medical Science, Fujian Medical University, Fuzhou, China; 3 Department of Obstetrics, Women and Children’s Hospital, School of Medicine, Xiamen University, Xiamen, China; 4 School of Public Health, Xiamen University, Xiamen, China

**Keywords:** *Group B Streptococcus*, Risk factors, Intrapartum antibiotic prophylaxis, Pregnant woman, Neonate

## Abstract

**Background:**

*Group B Streptococcus* (GBS), also referred as *Streptococcus agalactiae*, is one of the leading causes of life-threatening invasive diseases such as bacteremia, meningitis, pneumonia and urinary tract infection in pregnant women and neonates. Rates of GBS colonization vary by regions, but large-sample studies on maternal GBS status are limited in southern China. As a result, the prevalence of GBS among pregnant women and its associated risk factors and the efficacy of intrapartum antibiotic prophylaxis (IAP) intervention in preventing adverse pregnancy and neonatal outcomes remain poorly understood in southern China.

**Methods:**

To fill this gap, we retrospectively analyzed demographic and obstetrical data of pregnant women who have undergone GBS screening and delivered between 2016 and 2018 in Xiamen, China. A total of 43,822 pregnant women were enrolled and only a few GBS-positive women did not receive IAP administration. Possible risk factors for GBS colonization were assayed by univariate and multivariate logistic regression analysis. Generalized linear regression model was applicated to analyze whether IAP is one of the impact factors of the hospital length of stay of the target women.

**Results:**

The overall GBS colonization rate was 13.47% (5902/43,822). Although women > 35 years old (*P =* 0.0363) and women with diabetes mellitus (DM, P = 0.001) had a higher prevalence of GBS colonization, the interaction between ages and GBS colonization was not statistically significant in Logistic Regression analysis (adjusted OR = 1.0014; 95% CI, 0.9950, 1.0077). The rate of multiple births was significantly dropped in GBS-positive group than that of GBS-negative group (*P =* 0.0145), with no significant difference in the rate of fetal reduction (*P =* 0.3304). Additionally, the modes of delivery and the incidences of abortion, premature delivery, premature rupture of membranes, abnormal amniotic fluid and puerperal infection were not significantly different between the two groups. The hospitalization stays of the subjects were not influenced by GBS infection. As for neonatal outcomes, the cases of fetal death in maternal GBS-positive group did not statistically differ from that in maternal GBS-negative group.

**Conclusion:**

Our data identified that pregnant women with DM are at high risk of GBS infection and IAP is highly effective in prevention of adverse pregnancy and neonatal outcomes. This stressed the necessity of universal screening of maternal GBS status and IAP administration to the target population in China, and women with DM should be considered as priorities.

## Background

*Group B Streptococcus* (GBS), also known as *Streptococcus agalactiae*, is a Gram-positive bacterium which asymptomatically colonizes in women rectovaginal areas and could result in adult and neonatal invasive diseases under certain conditions [1]. GBS infection can lead to invasive diseases such as bacteremia and skin/soft tissue infection in nonpregnant adults, the burden of which has been increased significantly during the past few years [2–4]. The GBS carriage of pregnant women can be chronic, intermittent or transient and is implicated in urinary tract infection, premature rupture of membranes, and preterm birth [5–7]. According to a literature, the prevalence of GBS invasive diseases in pregnant women was twice as much as that in nonpregnant women [8]. GBS could be transmitted vertically from colonized mothers to their offspring through genital tract at or just before delivery, which may cause early-onset invasive neonatal GBS disease (EOD) that occurs < 7 days of life, often manifesting as bacteremia and pneumonia [9, 10]. The incidence of EOD among infants born to women with GBS colonization was 29 times higher than that of infants born to women without GBS colonization [11]. Invasive neonatal GBS disease that appears from 7 to 90 days of life is referred as late-onset disease (LOD), the common manifestations of which are bacteremia, urinary tract infection, and meningitis [12, 13]. Newborns with LOD are exposed to GBS by horizontal transmission. Although the risk factors of LOD are not as well understood as EOD, it was suggested that babies usually acquired the same serotype of GBS as their mothers’ colonized strains, and GBS-positive breast milk was implicated in heavy neonatal infection that GBS could be isolated from their throat, ear and rectum at least once [14].

It was reported by a meta-analysis that estimates of maternal GBS colonization in the world vary by regions, with rates ranging from 11 to 35% [15]. Annually, GBS infection causes high morbidity and mortality worldwide. The incidence rate of systemic invasive GBS diseases in pregnant women is 0.38 per 1000 pregnancies with case fatality rate of 0.2% [8]. The invasive GBS disease rate in newborns is 0.49 per 1000 live births [9]. The Centers for Disease Control and Prevention (CDC) recommends pregnant women at 35–37 weeks of gestation should be screened for GBS carriage through culture-based strategy or risk-based approach. And culture-positive women or women with any risk factors for EOD should receive intrapartum antibiotic prophylaxis (IAP) [16]. According to the recommendation provided by CDC, GBS-colonized parturient women were offered IAP at the time of labor onset or rupture of membranes until their delivery, and penicillin, ampicillin and cefazolin were currently the agents of choice for IAP.

In China, universal prenatal screening for GBS carriage has not been carried out. Given the regional variations in GBS infection, it is necessary to develop a proper strategy to test GBS colonization status in women at late pregnancy. Since there are no available GBS vaccines, it is essential to evaluate the efficacy of IAP in preventing GBS-related adverse outcomes at different regions [17, 18].We previously reported the combination of GBS chromogenic media with GBS carrot agar and β-γ detection agar enhanced the detection rate of GBS in vaginal and rectal swabs significantly [19], which was also used in this research to test GBS status among pregnant women. Wenjing Ji et al. found the incidence of invasive neonatal GBS diseases was 0.31 cases/1,000 live births and the case-fatality rate was 2.3% in China, suggesting enhanced surveillance and preventive strategies should be carried out in China [20]. Moreover, Yao Zhu’s group showed that IAP was effective in reducing GBS-EOD and recommended universal screening of maternal GBS and subsequent IAP intervention in China [11]. In this study, we aim to determine the prevalence of GBS among pregnant women in southern China, to find out the high risk-group of invasive GBS diseases and to calculate the efficacy of IAP treatment, which may aid in the development of intervention programs.

## Methods

### Population and Study Design

This study was carried out at Women and Children’s Hospital, School of Medicine, Xiamen University, China. Pregnant women who attended the hospital for prenatal tests including GBS screening and delivered from 2016 to 2018 were eligible for inclusion. Data on the subjects and their babies were obtained from their medical records and reviewed retrospectively. According to the algorithms provided by the CDC, women admitted with signs and symptoms of preterm labor and women with rupture of membranes at < 37 weeks and 0 days’ gestation should be collected vaginal-rectal swabs for GBS culture and started GBS prophylaxis once they entered true labor [16]. Therefore, a minority of the participants in this study with threatened preterm labor, risks of preterm delivery such as multiple births or premature rupture of membranes might have the test before 35 weeks of gestation. The antenatal screening results would guide GBS management of pregnant women at the time of labor. Since a negative GBS screen is considered valid for 5 weeks [16], the negative participants who did not give birth in 5 weeks would undergo repeat GBS screening before their delivery. This research was approved by the Human Research Ethics Committee of Women and Children’s Hospital of Xiamen University (KY-2020-103) and was conducted strictly in accordance with the approval.

### GBS colonization determination

GBS was tested as previously described [19]. The vaginal and rectal samples of each patient were collected using GBS TranSwab (Creative Lifesciences, China), which were subsequently incubated at 37 ℃ in 5% CO_2_ for 18 to 24 h within 2 h of collection. The detection of color change or red-orange pigment after enrichment was specific for the presence of β-hemolytic GBS strains. The negative samples (no color change or red-orange spots) were further inoculated onto GBS Carrot Agar and β-γ Detection Agar (Creative Lifesciences, China) and were cultivated for another 24 h in order to generate β-hemolysis in γ-hemolytic strains. The red-orange pigmented β-hemolytic colonies were indicative of nonhemolytic GBS strains, and the nonpigmented β-hemolytic colonies were finally detected by CAMP test or matrix-assisted laser desorption ionization–time of flight mass spectrometry (MALDI-TOF MS) to confirm the presence of nonpigmented or CAMP test-negative GBS strains. Once the pregnant women were confirmed GBS culture-positive, they would be provided with IAP.

### IAP administration

Pregnant women with threatened preterm labor, risks of preterm delivery such as multiple births or premature rupture of membranes could be collected vaginal-rectal swabs for GBS culture before 35 weeks of gestation. And all other pregnant women were screened for GBS at 35–37 weeks of gestation. At the time of labor or rupture of membranes, IAP should be given to women who tested positive for GBS colonization. If GBS culture results were unknown at the time of delivery onset, women at < 37 weeks and 0 days’ gestation, had a duration of membrane rupture ≥ 18 h, or had a temperature of ≥ 100.4º F (≥ 38.0ºC) were also treated with IAP. Once the GBS status were available prior to delivery and were negative, the GBS prophylaxis would be discontinued, otherwise IAP administration would be continued until their delivery. However, there were 90 culture-confirmed GBS-positive pregnant women were not administrated with IAP in time or more than 4 h. IAP agents and dosage were applied to patients according to the guidelines released by the CDC [16]. An initial dose of 4.8 million U of penicillin were given to GBS culture-positive women at the time of labor onset via intravenous injection, followed by 2.4 million U of penicillin at an interval of 4 h. Pregnant women with premature rupture of membranes were intravenously injected with an initial dose of 2 g ampicillin, followed by 1 g ampicillin at an interval of 4 h. Penicillin-allergic women could be administrated with cefazolin or clindamycin.

### Data abstraction and statistical analysis

The vaginal and rectal samples of each patient were collected followed consisted standard. The laboratory has passed ISO15189 quality management system certification. The medical record apartment has passed the five-level evaluation of the application level of the electronic medical record system of the National Health Commission of China. The general information and obstetrical data of the target population were entered into a standard Excel form and reviewed twice by the investigators, then we used R v4.0.5 software for statistical analysis. Normal distribution data were presented as $\stackrel{-}{x}$± s and skewness distribution data as M (P25, P75). Enumeration data were presented as absolute numbers. Z test or Mann-Whitney U test was employed for the comparison of measurement data between groups of women with GBS colonization vs. women without GBS colonization. Chi-square test or Fisher’s exact test was used for the comparison of enumeration data between groups of women with GBS colonization vs. women without GBS colonization. Generalized linear regression model was applicated to analyze the impact factors of the hospital length of stay of the target women. Logistic regression analysis model was used to assay the risk factors for GBS colonization and fetal distress. *P* < 0.05 (*) was considered statistically significant.

## Results

### Risk factors for GBS colonization among pregnant women

A total of 43,822 women were included in this study, with ages ranging from 16 to 56 years old. The demographic data of the investigated population were summarized in Table 1. The overall mean age of the 43,822 pregnant women was 30.67 ± 4.42 years old. Of them, about 13.46% (5902/43,822) were identified as GBS carriers. The correlation between age group and GBS colonization was statistically different (*P =* 0.0363). In detail, the proportion of GBS-positive women was higher in ≥ 35 years old group. The disparities in characteristics including ABO blood group and Rh blood group were statistically insignificant. Ethnic backgrounds or native places did not differ significantly between these two groups.

**Table 1:** Demographic characteristics of pregnant women

Variables		GBS colonization		*P* value
		Positive (n = 5902)	Negative (n = 37,920)	
Age (years)				0.0363*
	≤ 20	14 (0.24%)	138 (0.36%)	
	(20, 35)	4686 (79.40%)	30,501 (80.44%)	
	≥ 35	1202 (20.37%)	7281 (19.20%)	
ABO Blood Group				0.5703
	A	1666 (28.23%)	10,773 (28.41%)	
	B	1431 (24.25%)	9135 (24.09%)	
	O	2438 (41.31%)	15,481 (40.83%)	
	AB	367 (6.22%)	2531 (6.67%)	
Rh Blood Group				0.9309
	Negative	32 (0.54%)	209 (0.55%)	
	Positive	5870 (99.46%)	37,711 (99.45%)	
Ethnic Group				0.1774
	Han	5877 (99.58%)	37,715 (99.46%)	
	She	2 (0.03%)	31 (0.08%)	
	Miao	3 (0.05%)	25 (0.07%)	
	Hui	1 (0.02%)	25 (0.07%)	
	Mongolian	1 (0.02%)	23 (0.06%)	
	Other	18 (0.30%)	101 (0.27%)	
Native Place				0.1644
	Fujian	4924 (83.43%)	31,047 (81.88%)	
	Jiangxi	183 (3.10%)	1334 (3.52%)	
	Sichuan	87 (1.47%)	689 (1.82%)	
	Henan	76 (1.29%)	592 (1.56%)	
	Anhui	77 (1.30%)	473 (1.25%)	
	Other	555 (9.40%)	3785 (9.98%)	

The obstetrical data and underlying diseases of the study population were shown in Table 2. Women with threatened preterm labor, risks of preterm delivery such as multiple births or premature rupture of membranes might have the test before 35 gestational weeks. No significant difference was observed between GBS-positive women and GBS-negative women in terms of parity (*P =* 0.1688), gravidity (*P =* 0.4070) and gestational weeks (*P =* 0.6769). The proportion of pregnant women with diseases including anemia (*P =* 0.3946), eclampsia (*p =* 0.8879), cholestasis (*P =* 0.7179) and thyroid dysfunction (*P =* 0.8058) showed no significant difference between the two groups. Whereas the proportion of pregnant women with diabetes were significantly higher in GBS carriers than noncarriers (*P =* 0.0010).

**Table 2:** Obstetric characteristics of pregnant women

Characteristics		GBS colonization		*P* value
		Positive (n = 5902)	Negative (n = 37,920)	
Parity (times)				0.1688
	1	2097 (35.53%)	13,236 (34.91%)	
	2	1901 (32.21%)	11,968 (31.56%)	
	3	1119 (18.96%)	7294 (19.24%)	
	≥ 4	785 (13.30%)	5422 (14.30%)	
Gravidity (times)				0.4070
	0	254 (4.30%)	1773 (4.68%)	
	1	2835 (48.03%)	18,330 (48.34%)	
	2	2547 (43.15%)	16,237 (42.82%)	
	3	252 (4.27%)	1474 (3.89%)	
	≥ 4	14 (0.24%)	106 (0.28%)	
Gestational Age (weeks)				0.1571
	≤ 28	212 (3.59%)	1356 (3.58%)	
	(28, 32]	102 (1.73%)	830 (2.19%)	
	(32, 34]	118 (2.00%)	805 (2.12%)	
	(34, 37]	366 (6.20%)	2465 (6.50%)	
	> 37	5104 (86.48%)	32,464 (85.61%)	
Eclampsia				0.8879
	Yes	110 (1.86%)	693 (1.83%)	
	No	5792 (98.14%)	37,227 (98.17%)	
Cholestasis				0.7179
	Yes	114 (1.93%)	763 (2.01%)	
	No	5788 (98.07%)	37,157 (97.99%)	
Thyroid Dysfunction				0.8058
	Yes	501 (8.49%)	3179 (8.38%)	
	No	5401 (91.51%)	34,741 (91.62%)	
Diabetic Mellitus				0.0010^**^
	Yes	1233 (20.89%)	7230 (19.07%)	
	No	4669 (79.11%)	30,690 (80.93%)	

As indicated in Table 3, variables including ages and DM were further included in a multiple logistic regression analysis. After adjustment, only DM was significantly associated with GBS colonization (OR = 1.1175, 95% CI = 1.0423, 1.1973), while the relation between ages and GBS colonization was no longer statistically significant (OR = 1.0014, 95% CI = 0.9950, 1.0077).

**Table 3:** Logistic regression analysis of the risk factors for maternal GBS-colonization

Factors	β (SD)	95% CI of β	OR	95% CI of OR	*P* value
Ages	0.0014 (0.0032)	(-0.0050, 0.0077)	1.0014	(0.9950, 1.0077)	0.6724
Diabetes Mellitus	0.1111 (0.0354)	(0.0414, 0.1801)	1.1175	(1.0423, 1.1973)	0.0016**

Taken together, our data suggested that diabetic mellitus might be one of the risk factors for GBS colonization during pregnancy.

### Pregnancy outcomes of the Study Population by GBS colonization status

To evaluate the influence of IAP on pregnancy outcomes, we analyzed the rates of abortion and multiple-pregnancy between GBS carriers and negative controls and results were listed in Table 4. It seemed that the incidence of abortion (including spontaneous abortion and induced abortion) was similar in these two groups (*P =* 0.2084). In contrast, the proportion of multiple-pregnancy in GBS carriers was dropped significantly than that in GBS-negative group (*P =* 0.0145), with no significant difference in the rate of multifetal pregnancy reductions (*P =* 0.3304).

**Table 4:** Comparison of pregnancy outcomes between GBS carriers and noncarriers

Parameters		GBS colonization		*P* value
		Positive n = 5902	Negative n = 37,920	
Abortion				0.2084
	Yes	518 (8.78%)	3525 (9.30%)	
	No	5384 (91.22%)	34,395 (90.70%)	
Multiple-pregnancy				0.0145^*^
	Yes	170 (2.88%)	1332 (3.51%)	
	No	5732 (97.12%)	36,588 (96.49%)	
Multiple-pregnancy with fetal reduction				0.3304
	Yes	5 (2.94%)	66 (4.95%)	
	No	165 (97.06%)	1266 (95.05%)	

We further excluded 4019 women who did not deliver (ended their pregnancy with abortion or stillbirths). As shown in Table 5, the prevalence of GBS among pregnant women at different gestational ages was similar (*P =* 0.6769). The proportion of cesarean section in GBS-positive group did not differ from that in GBS-negative group significantly (*P =* 0.5573). In addition, there was no significant difference in terms of the rates of premature delivery (*P =* 0.2077), premature rupture of membranes (*P =* 0.6769) or abnormal amniotic fluid (*P =* 0.6634). Only one case of puerperal infection was found in GBS culture-negative pregnant women.

**Table 5:** The association between GBS colonization and adverse pregnancy outcomes

Factors		GBS colonization		*P* value
		Positive n = 5382	Negative n = 34,421	
Modes of Delivery				0.5573
	Vaginal Birth	3368 (62.58%)	21,687 (63.01%)	
	Cesarean Section	2014 (34.12%)	12,734 (36.99%)	
Premature Delivery				0.2077
	Yes	457 (8.49%)	3108 (9.03%)	
	No	4925 (91.51%)	31,313 (90.97%)	
Premature Rupture of Membranes				0.1282
	Yes	1164 (21.63%)	7129 (20.71%)	
	No	4218 (78.37%)	27,292 (79.29%)	
Abnormal amniotic fluid				0.6634
	Yes	180 (3.34%)	1195 (3.47%)	
	No	5202 (96.66%)	33,226 (96.53%)	
Puerperal Infection				1
	Yes	0	1	
	No	5382	34,420	

From these results, we concluded that though the multiple births rate was reduced among GBS-positive pregnant women, IAP intervention was important in ameliorating the adverse pregnancy outcomes including premature delivery, premature rupture of membranes, abnormal amniotic fluid and puerperal infection.

### Factors affecting the hospitalization stays of pregnant women analyzed by generalized Linear Regression Analysis

GBS related invasive diseases remains a heavy burden of public health system and the therapy of which costs a lot. Herein we used hospital length of stay as an indicator of disease severity and disease burden as Meredith Deutscher et al. suggested [21]. In order to evaluate the severity of GBS infection after IAP application, we assayed the effect of GBS carriage on the hospital length of stays of pregnant women by generalized linear regression analysis (Table 6). The data indicated that the hospitalization days have no association with GBS colonization status, ages of the pregnant women or occurrence of puerperal infection, but were positively related to cesarean section, gravidity, abortion, premature delivery, diabetes, eclampsia, anemia, thyroid dysfunction, cholestasis as well as multiple births, and were negatively related to parity and premature rupture of membranes.

**Table 6:** Generalized Linear Regression Analysis of the Hospital Length of Stays of Pregnant Women

Factors	β (SD)	95%CI	*P* value
GBS Colonization	0.049 (0.031)	(-0.012, 0.109)	0.1135
Ages	0.005 (0.003)	(-0.001, 0.010)	0.0796
Cesarean Section	1.868 (0.023)	(1.823, 1.914)	< 0.001^***^
Gravidity	0.062 (0.012)	(0.038, 0.084)	< 0.001^***^
Parity	-0.477 (0.025)	(-0.526, -0.428)	< 0.001^***^
Abortion	1.318 (0.288)	(0.753, 1.883)	< 0.001^***^
Premature Delivery	1.993 (0.043)	(1.908, 2.078)	< 0.001^***^
Diabetes Mellitus	0.190 (0.027)	(0.137, 0.244)	< 0.001^***^
Premature Rupture of Membranes	-0.197 (0.027)	(-0.249, -0.145)	< 0.001^***^
Eclampsia	0.416 (0.076)	(0.267, 0.565)	< 0.001^***^
Puerperal Infection	2.730 (2.091)	(-1.367, 6.828)	0.1916
Anemia	0.335 (0.024)	(0.288, 0.381)	< 0.001^***^
Thyroid Dysfunction	0.119 (0.038)	(0.045, 0.193)	0.0017^**^
Cholestasis	0.983 (0.080)	(0.457, 0.735)	< 0.001^***^
Multiple-pregnancy	0.596 (0.071)	(0.648, 0.921)	< 0.001^***^

These results revealed that after IAP therapy, the hospitalization stays of pregnant women were not affected by GBS infection.

### Outcomes of newborns by maternal GBS colonization status

Of the 40,905 fetuses carried by the study population, 81 were stillbirths. As shown in Table 7, the incidence of stillbirth between maternal GBS-colonized group and noncolonized group did not differ significantly (*P =* 0.8975). In order to figure out the effect of IAP application to GBS carriers on infants, we examined the characteristics of the remaining 40,824 neonates and summarized in Table 8. No significant difference was observed in fetal gender, weight, height or the rate of nuchal cord or cord torsion (*P =* 0.6284) between maternal GBS-positive group and maternal GBS-negative group. Although the APGAR score was comparable in these two groups, the incidence of fetal distress in maternal GBS-colonized group was significantly declined than that in noncolonized group (*P =* 0.0385), which was out of our expectation.

**Table 7:** The incidence of stillbirth in maternal GBS-positive group and maternal GBS-negative group

		Maternal GBS Colonization		*P* value
		Positive n = 5502	Negative n = 35,403	
Stillbirth	Yes	10 (0.18%)	71 (0.20%)	0.8975
	No	5492 (99.82%)	35,332 (99.80%)	

**Table 8:** The basic information of live birth infants

Characteristics		Maternal GBS Colonization		*P* value	
		Positive n = 5492	Negative n = 35,332		
Fetal Gender				0.1213	
	Male	2923 (53.22%)	19,204 (54.35%)		
	Female	2569 (46.78%)	16,128 (45.65%)		
Weight (grams)		3195.96 ± 479.79	3184.97 ± 497.77	0.1163	
Height (centimeters)		49.58 ± 1.97	49.56 ± 1.94	0.4375	
Nuchal Cord or Cord Torsion				0.6284	
	Yes	1848 (33.65%)	12,010 (33.99%)		
	No	3644 (66.35%)	23,322 (66.01%)		
Fetal Distress				0.0385^*^	
	Yes	302 (5.50%)	2201 (6.23%)		
	No	5190 (94.50%)	33,131 (93.77%)		
APGAR1		9.87 ± 0.55	9.87 ± 0.59	0.3487	
APGAR5		9.93 ± 0.36	9.92 ± 0.39	0.7426	
APGAR10		9.94 ± 0.31	9.94 ± 0.35	0.6973	

This finding prompted us to re-analyze our data with mothers taken as the study subjects. As summarized in Table 9, the differences between pregnant women with fetal distress and pregnant women without distress were statistically significant in ages (*P* < 0.001), gestational ages (*P* < 0.001), the rate of multiple pregnancies (*P* < 0.001), the incidence of nuchal cord or cord torsion (*P* < 0.001) as well as abnormal amniotic fluid (*P* < 0.001). However, no significant difference was found between these two groups in terms of the incidences of cholestasis (*P =* 0.5967), diabetic mellitus (*P =* 0.0915) and GBS colonization (*P =* 0.4841). We further assayed the potential impact factors of fetal distress in a logistic regression model. The results in Table 10 indicated that among the factors, ages, gestational ages and the occurrence of nuchal cord or cord torsion and abnormal amniotic fluid were significant. The risk of fetal distress ascended with decreasing ages (OR = 0.9673, 95% CI=-0.046, -0.0204), and descended with increasing gestational ages (OR = 1.0997, 95% CI = 1.0712, 1.1312) and the occurrence of nuchal cord or cord torsion (OR = 2.1794, 95% CI = 1.9587, 2.4252) and abnormal amniotic fluid (OR = 3.8839, 95% CI = 3.2448, 4.6204). All other factors including multiple-pregnancy, cholestasis, diabetes mellitus and GBS colonization were found to be insignificant.

**Table 9:** Maternal characteristics by fetal distress

Characteristics		Fetal Distress		*P* value	
		Yes n = 1436	No n = 42,386		
Age (Years)		29.91 ± 4.25	30.63 ± 4.42	< 0.001***	
Gestational age (weeks)		39.71 (38.57, 40.43)	39.14 (38.14, 40.00)	< 0.001***	
Multiple births				< 0.001***	
	Yes	22 (1.53%)	1480 (3.49%)		
	No	1414 (98.47%)	40,906 (96.51%)		
Cholestasis				0.5967	
	Yes	32 (2.23%)	845 (2.00%)		
	No	1404 (97.77%)	41,541 (98.01%)		
Diabetic Mellitus				0.0915	
	Yes	252 (17.55%)	8211 (19.37%)		
	No	1184 (82.45%)	34,175 (80.63%)		
Nuchal Cord or Cord Torsion				< 0.001***	
	Yes	736 (51.25%)	13,032 (30.75%)		
	No	700 (48.75%)	29,354 (69.25%)		
Abnormal amniotic fluid				< 0.001***	
	Yes	158 (11.00%)	1307 (3.08%)		
	No	1278 (89.00%)	41,079 (96.92%)		
GBS colonization				0.4841	
	Yes	184 (12.81%)	5718 (13.49%)		
	No	1252 (87.19%)	36,668 (86.51%)		

**Table 10:** Logistic regression analysis of the risk factors for fetal distress

Factors	β (SD)	95% CI of β	OR	95% CI of OR	*P* value
Ages	-0.0332 (0.0066)	(-0.046, -0.0204)	0.9673	(0.9549, 0.9798)	< 0.001***
Gestational ages	0.0950 (0.0139)	(0.0687, 0.1233)	1.0997	(1.0712, 1.1312)	< 0.001***
Multiple-pregnancy	-0.4253 (0.2221)	(-0.8896, -0.0148)	0.6535	(0.4108, 0.9853)	0.0555
Cholestasis	0.3176 (0.1850)	(-0.0644, 0.6630)	1.3738	(0.9376, 1.9407)	0.0859
Diabetes Mellitus	0.0187 (0.0726)	(-0.1255, 0.1592)	1.0188	(0.8820, 1.1726)	0.7972
Nuchal Cord or Cord Torsion	0.7790 (0.0545)	(0.6723, 0.8859)	2.1794	(1.9587, 2.4252)	< 0.001***
Abnormal amniotic fluid	1.3568 (0.0901)	(1.1771, 1.5305)	3.8839	(3.2448, 4.6204)	< 0.001***
GBS colonization status	0.0512 (0.0808)	(-0.1044, 0.2125)	1.0525	(0.9008, 1.2368)	0.5263

Overall, our research suggested that IAP treatment was highly effective in preventing adverse neonatal outcomes. Although the fetal distress rate of babies born to GBS carriers was reduced in comparison with non-carriers, the logistic regression analysis showed the relation between fetal distress and GBS colonization was insignificant.

## Discussion

The current study suggested that the prevalence of GBS colonization was 13.47% (5902/43,822) in Xiamen, China. We demonstrated that in ≥ 35 years old pregnant women, the proportion of GBS carriers was significantly higher than non-carriers. Whereas the association between ages and GBS colonization was not statistically significant. In the contrast, DM is one of the risk factors for GBS colonization. After IAP usage, pregnancy and neonatal outcomes and hospitalization of stays were not affected by GBS status.

The regional difference of GBS colonization rate has been verified by lots of researches. Others reported 4.9% in Shenzhen, China [22], 17% in Karachi, Pakistan [23], 6.5% in Kocaeli, Turkey [24], 4.2 to 28.4% in Brazil [25] and 21% in the Hague, The Netherlands [26]. The disparities in sampling sites, detection methods and target populations might attribute to the regional variations. It stressed the necessity to promote universal GBS screening at different regions.

Consisted with our finding, Tsering Chomu Dechen et al. showed that the relation between age group and GBS culture positivity was statistically insignificant [27]. While an early study has indicated the statistically significant association between increasing ages and lower GBS culture-positive rate [28], which was contrary to our conclusion. Since some studies have reported the correlation of GBS detection and decreased α-diversity and Lactobacillus species, we reckoned the impaired vaginal microecology in elder pregnant women might account for the increasing GBS detection rate [29, 30]. If an association between maternal age and GBS colonization exists in the southern of China, it might be swamped by other variables in our research. Several studies have claimed that GBS were highly invasive in diabetic patients [4, 31], which supported our observation. There were also some studies declaring the significant association between increasing parity, gravidity and gestational ages and higher GBS colonization rate [22, 32, 33]. The increased susceptibility to GBS of these women might result from their decreased immunity and ability to eliminate the organism. Many studies have showed the significantly higher burden of GBS invasive diseases in black race than nonblack women [7, 34]. Furthermore, a study focused on a multicultural pregnant population from the Netherlands, showing that compared to European women, African women were at higher risk for GBS carriage while Asian women at lower risk [26]. However, the reason for these differences remains elusive. To our knowledge this is the first report to identify the rate of multiple births was significantly dropped in GBS-positive group than that in GBS-negative group, with no significant difference in the rate of multifetal pregnancy reductions. But further studies are needed to examine the underlying mechanism.

Once they were confirmed GBS culture-positive, the pregnant women would be offered IAP based on the guideline developed by the CDC. Our data revealed that after IAP usage, the pregnancy outcomes were not significantly influenced by GBS infection. And the hospital length of stay of pregnant women was not correlated to GBS status, suggesting IAP is effective in preventing severe GBS invasive diseases and reducing the disease burden to GBS-positive pregnant women and the public health system. An earlier study focused on pregnant women who were not on IAP intervention at the third trimester showed that GBS infection was significantly associated with premature delivery and premature rupture of membranes [27]. And the finding that GBS colonization rate was significantly reduced in penicillin G-treated group than that in untreated-group also supports our conclusion [35].

As to neonatal outcomes, we did not directly investigate the correlation between GBS carriage and invasive neonatal GBS diseases due to the lack of the relevant data, being one of the main flaws of this study. However, we found the fatality rate, gender, weight, height, APGAR scores and the rates of nuchal cord or cord torsion were not affected by maternal GBS colonization. While the incidence of fetal distress was significantly declined by maternal GBS colonization, which might result from some cofounders since no significant association between GBS colonization and fetal distress was observed in logistic regression analysis. In contrast, elevated risk of fetal distress was significantly correlated with decreasing ages, increasing gestational weeks as well as the occurrence of nuchal cord or cord torsion and abnormal amniotic fluid. There was a report demonstrated that IAP resulted in a 50% decrease in the occurrence of GBS-associated neonatal sepsis [36]. Another research clarified that standard IAP was a protective factor for GBS-EOD by logistic regression analysis [11]. Additionally, the incidence of EOD-GBS was reduced from 0.7 cases/1,000 live births in 1997 to 0.21–0.25 cases/1,000 live births in 2014 and 2015 [1]. These studies, in concert with our research, suggested that IAP usage was highly effective in preventing newborns from adverse outcomes including GBS invasive diseases.

According to the guideline released by the CDC, the strategies for GBS screening and IAP intervention based on risk factors or antenatal universal GBS-culture were available [16]. Whereas the comparison of risk factors versus intrapartum culture screening indicated that the latter was more precise in detecting GBS and was optimal in guiding IAP therapy [18]. Herein we described the prevalence of GBS in pregnant women at late pregnancy was 13.47% based on the culture method. Additionally, we found that women with diabetics were more susceptible to GBS colonization. Though IAP usage was effective in preventing pregnant women and newborns from adverse outcomes, there were still 90 culture-confirmed GBS-positive pregnant women in this study were not administrated with IAP in time or more than 4 h. It underlies the urgent need for rapid tests to detect GBS status. Laboratory detection of GBS takes a lot of time to culture GBS, ranging from 24 to 72 h. To solve this problem, we are now researching a new fluorescent immunochromatographic GBS antigen detection kit, aiming to shorten the testing time to 4–6 h. We hope this detection kit could be applied to clinical practice in future and help to improve the efficacy of IAP treatment. The high prevalence of GBS colonization and the high efficacy of IAP protection has underlined the need for GBS universal screening and IAP application for the target population in China.

## Data Availability

The original data presented in the study are included in the article/Supplementary Material. Further inquiries can be directed to the corresponding authors.

## Abbreviations

GBS: Group B streptococcus
IAP: Intrapartum antibiotic prophylaxis
DM: Diabetes mellitus
EOD: Early-onset disease
LOD: Late-onset disease
CDC: The Centers for Disease Control and Prevention
